# Tooth Enamel Demineralization: Caries and Erosion from the Viewpoint of Chemistry

**DOI:** 10.3390/dj14060387

**Published:** 2026-06-22

**Authors:** Joachim Enax, Erik Schulze zur Wiesche, Matthias Epple

**Affiliations:** 1 Research Department, Dr. Kurt Wolff GmbH & Co. KG, Johanneswerkstr. 34–36, 33611 Bielefeld, Germany; joachim.enax@drwolffgroup.com (J.E.); erik.schulze-zur-wiesche@drwolffgroup.com (E.S.z.W.); 2 Inorganic Chemistry and Center for Nanointegration Duisburg-Essen (CENIDE), University of Duisburg-Essen, Universitaetsstr. 5–7, 45117 Essen, Germany

**Keywords:** demineralization, caries, erosion, erosive tooth wear, calcium phosphate, hydroxyapatite, pellicle, plaque, enamel

## Abstract

The demineralization of tooth enamel is the primary consequence of dental caries, leading to cavities and finally tooth loss. Erosive tooth wear from acidic beverages and food is another factor that degrades enamel. In both cases, an acidic environment leads to etching and the final dissolution of tooth mineral, i.e., hydroxyapatite. Here, this process is discussed from a chemical perspective, taking into account the solubility of calcium phosphate and the presence of the pellicle (protein layer) and plaque (bacterial biofilms), which both affect the dissolution rate. While low pH is definitely decisive, calcium-binding ligands (e.g., acid anions, proteins) contribute to dissolution by removing calcium ions from the equilibrium. This is an important effect in the oral cavity where the concentration of biomolecules is high. The situation is complicated by the fact that the composition of saliva and the oral microbiome vary considerably between individuals. The state of current knowledge on the demineralization of enamel is summarized and discussed, also in the context of approaches to prevent dental caries and erosive tooth wear.

## 1. Introduction

In dentistry, the term demineralization denotes the loss of mineral from enamel and dentin due to acid challenges, i.e., a low pH. It represents the initial event in caries, causing a local demineralization that is visible as a white spot on the enamel surface. Erosive tooth wear denotes the surface demineralization caused by acidic beverages and food. A sound understanding of the physicochemical mechanisms underlying mineral loss is important for preventive protocols and the evaluation of potential remineralization therapies.

At first glance, from the viewpoint of chemistry, the demineralization of tooth enamel is a simple, seemingly trivial process. Enamel consists most prominently of calcium phosphate, i.e., hydroxyapatite, which is an acid-soluble inorganic salt. Its solubility is governed by the solubility product and therefore depends on the composition of the surrounding medium. Its dissolution is driven mostly by undersaturation in the surrounding medium, which is mainly a question of a low pH. Apparently, there should not be much to discuss if there is a pH drop caused by acidic beverages or by acid-producing cariogenic bacteria. We will demonstrate in the following sections that the underlying processes are much more complex than that and, realistically speaking, are not yet fully understood despite decades of intense research. We will also distinguish between the driving force for enamel dissolution (basically governed by thermodynamics) and the rate of dissolution (basically driven by kinetics).

## 2. Demineralization Mechanisms

Two major mechanisms of enamel dissolution in-vivo can be distinguished. Caries involves the acidic attack by organic acids that are formed by bacterial biofilms directly on the tooth surface [1]. Erosion denotes the dissolution of enamel by acidic beverages and food. As a rule, erosion is easier to understand and to replicate than caries.

Acidic beverages and food decrease the local pH at the tooth surface and cause enamel dissolution. Typical pH values of such erosive beverages and food are around 2.5 to 3.5 [2]. Gastric acid, with a pH of about 1, is the most corrosive liquid that can be present in the mouth (e.g., after reflux or bulimia) [3]. It should be emphasized that the pH scale is logarithmic, i.e., a solution with pH 2.5 contains 10^1^ = 10 times more protons (as H_3_O^+^) than one with pH 3.5. Erosion leads to defects and material loss in enamel, i.e., it starts at the tooth surface and progresses towards the dentin. In the following, we define “erosion” (as abbreviated from “dental erosion”) as the chemical process of acidic attack without additional mechanical stress. We further define “erosive tooth wear” as the combination of acidic attack and mechanical stress that together induce enamel abrasion, promoted by enamel softening after acidic attack [4].

Basically, caries relies on the same mechanism as erosion, but the acid is not delivered by food or beverages but produced inside a biofilm in direct contact with enamel. Fermentable carbohydrates are metabolized by oral biofilms, and strains like *Streptococcus mutans*, *Lactobacillus* spp., and *Actinomyces* spp. produce organic acids like lactic acid or acetic acid that lower the local pH and dissolve tooth mineral directly on the tooth surface, where the buffering and remineralizing effect of saliva is restricted due to the dense biofilm. The pH in a cariogenic biofilm is around 4.3, i.e., well below the critical pH of enamel (about 5.5) that is usually assumed for enamel dissolution [5].

## 3. The Structure of Enamel

In the following, we briefly summarize the structure of enamel, as this is essential to understanding its demineralization. The structure of human teeth has been extensively researched and discussed (see, e.g., refs. [6,7,8,9] for review articles and refs. [10,11] for classical books on this subject). First of all, a tooth is a well-structured biomineralized hard tissue and not just a cm-sized crystal of an inorganic salt. In humans and many mammals, the tooth surface consists of enamel containing approximately 96% inorganic mineral by weight, organized in a rod-like microstructure. Tooth mineral consists of calcium phosphate in the form of hydroxyapatite rods, Ca_5_(PO_4_)_3_OH. Hydroxyapatite is the most prominent member of the family of calcium phosphates that occur in different stoichiometries with individual solubility [12,13]. In humans and mammals, hydroxyapatite is the mineral phase of teeth and bones. It is usually denoted as “biological apatite” or “bioapatite” because it always occurs as a non-stoichiometric phase where the constituting ions Ca^2+^, PO_4_^3−^, and OH^−^ are substituted by other ions. Biological apatite contains a small percentage of substituting ions like carbonate CO_3_^2−^, hydrogen phosphate HPO_4_^2−^, and magnesium Mg^2+^ that affect the mineral solubility in acids [14]. The apatite rods in enamel are elongated along the crystallographic *c*-axis in the hexagonal crystal system. Interprismatic spaces between the hydroxyapatite rods facilitate diffusion and influence the kinetics of mineral exchange. In contrast, the underlying dentin contains about 70% mineral by weight and consists of a collagen-rich organic matrix with radial tubules that permit permeability, diffusion, and fluid transport.

Chemically and structurally, the outer surface of a tooth is less ordered than the interior because the surface experiences a continuous cycle of demineralization and remineralization, leading to an unstructured layer of calcium phosphate. Inside the tooth, the well-oriented hydroxyapatite rods form the solid basis of enamel. It is conceivable that the disordered surface has a higher kinetic and thermodynamic solubility than the intact enamel below, just by its poorly crystalline and partially amorphous nature. Furthermore, the enamel rods are protected by the enclosing proteins, i.e., the organic matrix that remained from the biomineralization process during tooth formation. Besides the presence of proteins, the chemical composition of enamel at the surface of a tooth is not much different from the enamel below the surface.

Here, we will consider only the demineralization of the tooth surface, i.e., of enamel. The acidic degradation of enamel is easier to understand than that of dentin because the fraction of organic matrix is much lower in enamel than in dentin; therefore, we can focus our attention on the enamel mineral and mostly ignore the proteins inside the enamel.

## 4. The Solubility Product of Hydroxyapatite and Enamel

Hydroxyapatite is usually formulated as Ca_5_(PO_4_)_3_OH. In general, hydroxyapatite is almost insoluble in water at neutral pH but (like all calcium phosphates) soluble in acids, which is the underlying cause for its sensitivity towards erosive tooth wear and caries. The solubility of hydroxyapatite is defined by its solubility product *L* and the ion product *IP*:

*L* = [Ca^2+^]^5^ · [PO_4_^3−^]^3^ · [OH^−^] (1)

(ion concentrations of a solution that is in equilibrium with solid hydroxyapatite)

and

*IP* = [Ca^2+^]^5^ · [PO_4_^3−^]^3^ · [OH^−^] (2)

(actual ion concentrations in a given solution).

**Figure 1:**
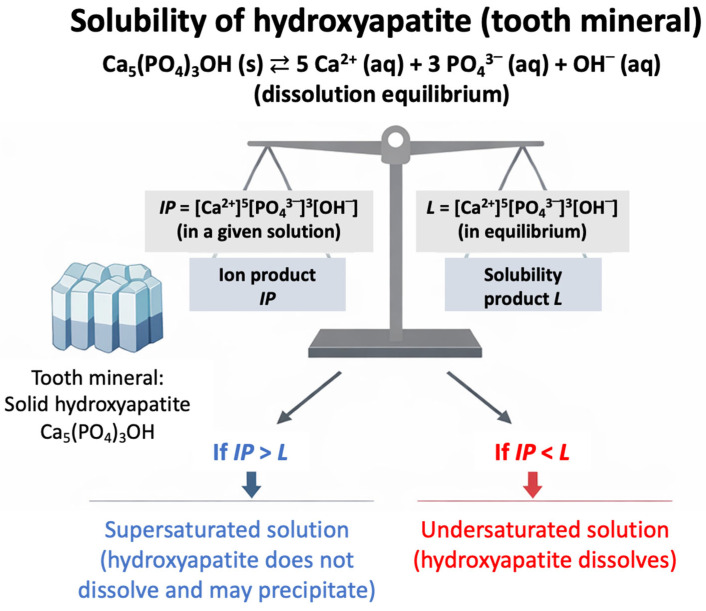
Solubility equilibrium of hydroxyapatite.

This gives the thermodynamic reason for the dissolution of hydroxyapatite [15]. If the actual product of the ion concentrations in the surrounding solution (the ion product *IP*) is smaller than *L*, the solution is undersaturated and hydroxyapatite will dissolve. The formula Ca_10_(PO_4_)_6_(OH)_2_ that is also sometimes used in the literature represents the number of atoms per crystallographic unit cell, which is twice the standard formula for hydroxyapatite. These two formulae can be easily converted by taking the double molar mass for the second formula. However, this affects the value of the solubility product *L*, which is about 10^−55^ mol^9^ L^−9^ for Ca_5_(PO_4_)_3_OH and 10^−110^ mol^18^ L^−18^ for Ca_10_(PO_4_)_6_(OH)_2_ (note the different units). Figure 1 illustrates the concept of the solubility equilibrium.

The definition of the solubility product leads to the apparently simple approach to measure the concentrations of calcium, phosphate, and hydroxide (via the pH value) in a given solution and to compute the ion product. Unfortunately, the situation is more complicated, and this simple approach leads to a serious miscalculation and an overestimation of *IP*.

It is not correct to insert the total phosphate concentration into the balance for *IP*. Instead, the concentration of fully deprotonated orthophosphate PO_4_^3−^ must be used (Equation (1)). Phosphoric acid has three deprotonation steps with the three acid dissociation constants pK_a_ of 2.14, 7.20, and 12.37. Thus, orthophosphate is present to nearly 100% only at a pH > 13. At all lower pH values, there are different protonation steps to hydrogenphosphate, dihydrogenphosphate, and even phosphoric acid, meaning that only a fraction of phosphate is present as orthophosphate. Besides the protonation of OH^−^, the main reason for the increased solubility of hydroxyapatite at decreasing pH is the associated protonation of phosphate species, which decreases the effective orthophosphate concentration even at constant total phosphate concentration. The concentrations of the different phosphate species at variable pH are generally not measured but computed, based on the three acid dissociation constants pK_a_ of phosphoric acid.

PO_4_^3−^ + H_3_O^+^ ⇄ HPO_4_^2−^ + H_2_O  (pK_a3_ = 12.37) (3)

HPO_4_^2−^ + H_3_O^+^ ⇄ H_2_PO_4_^−^ + H_2_O  (pK_a2_ = 7.20) (4)

H_2_PO_4_^−^ + H_3_O^+^ ⇄ H_3_PO_4_ + H_2_O  (pK_a1_ = 2.14) (5)

with the total concentration of phosphate

[phosphate] = [PO_4_^3−^] + [HPO_4_^2−^] + [H_2_PO_4_^−^] + [H_3_PO_4_] (6)

Consequently, the supersaturation of saliva is strongly overestimated if the total phosphate concentration is introduced into *IP*. At pH 7 and [phosphate] = 5.1 × 10^−3^ M (conditions in saliva), the concentration of [PO_4_^3−^] is about 2.55 × 10^−8^ M, i.e., only 1/200,000 of [phosphate] [16]. As the concentration of phosphate is to the third power in the solubility product, the error is (2 × 10^5^)^3^ = 8 × 10^15^, i.e., the supersaturation would be extremely overestimated if the total phosphate concentration was introduced. Finally, for a correct physicochemical treatment, the ion concentrations have to be converted to activities (see refs. [17,18] for the corresponding activity coefficients that range from 0.02 to 1), a fact which is usually ignored.

The demineralization of enamel can be followed indirectly, usually by measuring the increasing calcium and/or phosphate concentration or the consumption of acid in pH-stat experiments. However, the computation of the amount of dissolved mineral by measuring the amount of added base to keep the pH constant is also not as straightforward as it appears, as we will demonstrate in the following [19].

If we assume that enamel mineral consists of pure hydroxyapatite, it releases orthophosphate ions during dissolution. These will be protonated to a variable degree, depending on the pH. For instance, at the third pK_a_ value of phosphoric acid of 2.14, we have a 1:1 mixture of H_2_PO_4_^−^ and H_3_PO_4_. In that case, each phosphate group consumes on average 2.5 H_3_O^+^ ions, leading to 3 × 2.5 + 1 = 8.5 H_3_O^+^ ions per hydroxyapatite formula unit (the hydroxide group OH^−^ consumes another H_3_O^+^ ion). If hydroxyapatite is dissolved at pH 5, we have almost exclusively H_2_PO_4_^−^, i.e., 7 H_3_O^+^ ions per formula unit.

It must also be stressed that values given for the solubility product of tooth mineral are generally based on the stoichiometric formula of hydroxyapatite given as Ca_5_(PO_4_)_3_OH. The fact that tooth mineral (bioapatite) is not stoichiometric because it contains hydrogen phosphate and carbonate instead of orthophosphate, compensated by a deficit in calcium, is often not considered. A typical formula for a calcium-deficient hydroxyapatite that contains hydrogen phosphate is Ca_5−x/2_(PO_4_)_3−x_(HPO_4_)_x_OH. A typical formula for a calcium-deficient hydroxyapatite that contains carbonate is Ca_5−x/2_(PO_4_)_3−x_(CO_3_)_x_OH. In reality, both ionic substitutions occur, leading to very complex overall stoichiometries of tooth mineral and consequently to a more complex formula for the solubility product. In fact, this is almost never taken into account [20]. In terms of acid consumption, we obtain the following equations that highlight the complexity of enamel demineralization:

Ca_5_(PO_4_)_3_OH + **10 H_3_O**^+^ → 5 Ca^2+^ + 3 H_3_PO_4_ + 11 H_2_O (at pH 1) (7)

Ca_5_(PO_4_)_3_OH + **8.5 H_3_O^+^** → 5 Ca^2+^ + 1.5 H_3_PO_4_ + 1.5 H_2_PO_4_^−^ + 8 H_2_O (at pH 2.14) (8)

Ca_5_(PO_4_)_3_OH + **7 H_3_O^+^** → 5 Ca^2+^ + 3 H_2_PO_4_^−^ + 8 H_2_O (at pH 5) (9)

Ca_5_(PO_4_)_3_OH + **4 H_3_O^+^** → 5 Ca^2+^ + 3 HPO_4_^2−^ + 5 H_2_O (at pH 10) (10)

Ca_5−x/2_(PO_4_)_3−x_(HPO_4_)_x_OH + **(10 − x) H_3_O^+^** → (5 − x/2) Ca^2+^ + 3 H_3_PO_4_ + (11 − 1.5x) H_2_O (at pH 1) (11)

Ca_5−x/2_(PO_4_)_3−x_(HPO_4_)_x_OH + **(7 − x) H_3_O^+^** → (5 − x/2) Ca^2+^ + 3 H_2_PO_4_^−^ + (8 − 1.5x) H_2_O (at pH 5) (12)

Ca_5−x/2_(PO_4_)_3−x_(CO_3_)_x_OH + **7 H_3_O^+^** → (5 − x/2) Ca^2+^ + (3 − x) H_2_PO_4_^−^ + x CO_2_ + (8 + x) H_2_O (at pH 5) (13)

Depending on the pH and the composition of the apatite, between 4 and 10 H_3_O^+^ ions are necessary to dissolve one formula unit of hydroxyapatite. Other calcium phosphate phases may also occur, e.g., by reprecipitation during the dissolution process, making accurate measurements and predictions very complicated [21].

The substitutions in bioapatite are important because the presence of carbonate has been shown to increase the solubility of biological apatite, e.g., when bone substitution materials are resorbed by osteoclasts [22,23]. The solubility product of a carbonated apatite with the formula Ca_5−x/2_(PO_4_)_3−x_(CO_3_)_x_OH would be defined as

*L* = [Ca^2+^]^5−x/2^ · [PO_4_^3−^]^3−x^ · [CO_3_^2−^]^x^ · [OH^−^] (14)

The sum of the exponents is not 9 but 5 − x/2 + 3 − x + x + 1 = 9 − x/2, making the value of this solubility product not comparable with that of hydroxyapatite due to different units, i.e., mol^9−x/2^ L^−9+x/2^ vs. mol^9^ L^−9^. In the given case, the whole protonation equilibrium of carbonate (CO_3_^2−^) ⇄ hydrogen carbonate (HCO_3_^−^) ⇄ carbonic acid (H_2_CO_3_) would also come into play. The presence of other ions like fluoride or magnesium in the solid calcium phosphate would all enter the solubility product with their concentrations to the power of the stoichiometric coefficient.

Early constant composition studies by Nancollas and Chen have shown small but significant differences between bovine and human enamel in terms of the dissolution rate. At pH 4.5, bovine enamel dissolved more slowly, in line with a lower solubility product (5.4 × 10^−53^ vs. 3.9 × 10^−55^ mol^9^ L^−9^) [19]. However, it is unclear how the solubility product was defined for enamel as it is not a stoichiometric solid. Hassanali et al. have proposed a new value for the solubility product of enamel in 2017 with 10^−121^ (no unit given), based on the demineralization rate [24]. However, both reports can serve as an example for potential pitfalls during solubility studies. First of all, the solubility product must be defined according to a chemical formula, as otherwise it has no meaning. The solubility product is never dimensionless but always has the unit mol*^n^* L^−*n*^ with *n* the number of ions in the formula of the solid. If the stoichiometry of hydroxyapatite is assumed for enamel, erroneous conclusions result if [Ca^2+^], [PO_4_^3−^] and [OH^−^] are taken from experimental observations. The ratio [Ca^2+^]/[PO_4_^3−^] = 1.67 is also not applicable for the solubility product of enamel because this is not the case for the calcium-deficient bioapatite with [Ca^2+^]/[PO_4_^3−^] < 1.67 in enamel mineral [13]. We conclude that because the stoichiometry of enamel mineral is not known, it does not make sense to give a solubility product for enamel.

## 5. Demineralization of Enamel in the Presence of Organic Molecules, Proteins, and Bacteria

Besides a low pH, the dissolution of enamel is chemically enhanced by the presence of compounds that form a complex with calcium ions by coordinating bonds. This directly decreases the calcium concentration in the solubility product and can lead to dissolution at a higher pH than expected. A well-known example from general chemistry is ethylenediaminetetraacetic acid (EDTA), which forms a very stable calcium complex. In the mouth, typical complexing agents are lactate and citrate anions and proteins that can bind calcium and decrease the calcium concentration in the solubility product [18]. This was quantitatively demonstrated as early as 1966 by Gray, who showed that not only a low pH but also the total concentration of the attacking weak organic acid (here: lactic acid) is important for the dissolution of enamel.

Unfortunately, even under strictly controlled chemical conditions and in the absence of biomolecules like proteins, the solubility equilibrium of tooth mineral is complex and not directly accessible without a computer program that takes into account all equilibria that occur simultaneously. Such numerical analyses need exact data on solubility products, complex binding constants, and acid constants of phosphoric acid, carbonic acid, and of organic acids that are not always known with sufficient accuracy [18].

The presence of biomolecules adds to the complexity. To quote from Flemming et al. (2022) on the demineralization of enamel [25]: “*At first glance, it seems as if these topics have been explored completely and are the content of textbooks. A closer look at different aspects indicates that several key features are still not fully understood. This applies especially for the interactions of organic and inorganic structures directly underneath the surface.*” We note that many of the early studies on demineralization considered only the chemical part of the system, i.e., inorganic and organic ions, but did not take the biological agents into account. This started around 1980 (see also the bibliometric section below).

A numerical prediction is not possible in the presence of biomolecules of mostly unknown nature and concentration. For a strict computation, data like binding constants between calcium ions and individual proteins would be required, but these are often unknown. Besides calcium, phosphate is less prone to be removed from the solution by binding to chemical compounds, but binding to proteins is well possible. This underscores the need for experimental studies in a situation that cannot be assessed theoretically.

The pellicle denotes the thin layer of various proteins on the surface of teeth that forms within seconds from saliva [1]. Hannig has shown that it has a thickness of 10–20 nm after 1 min in contact with saliva that increases to several 100 nm within the next hours [26]. Although its composition has been determined by proteomics and other techniques [25,27], it is very difficult to quantitatively assess its effect on enamel dissolution. There is a broad consensus that the pellicle usually inhibits demineralization [25]. Flemming et al. have presented a thorough literature survey on the effect of the pellicle on demineralization and remineralization. They concluded that the pellicle represents a double-edged sword: it can prevent demineralization by protecting the enamel surface from acids but it can also prevent remineralization by forming a coating on the enamel surface that constrains the diffusion of calcium and phosphate ions [25]. They also stated that a full remineralization of caries lesions as proposed previously [20] is not possible because of the immediate and irreversible absorption of the pellicle proteins on enamel inside the cavity [25].

Thus, the pellicle plays an important role with respect to remineralization [25,28]. Flemming et al. have pointed out that the composition of the pellicle is different among individual persons [25]. It remains, however, unclear whether this affects the caries-activity of individuals. No significant differences in the pellicle proteins were found by Trautmann et al. by proteomics between caries-active and caries-inactive individuals [29].

Stephan postulated in the 1940s that the pH drop inside plaque in contact with enamel leads to caries and enamel dissolution. The time-dependent drop in pH after sugar intake is known as the so-called “Stephan Curve”. A “critical pH” between 5 and 5.5, depending on the individual, was formulated to assess the effect of a pH drop on enamel dissolution [30,31,32]. However, these fundamental results were critically discussed in later decades when it became obvious that the pH inside plaque is not easily measured and sometimes is even inhomogeneous. Dawes discussed the concept of critical pH in 2003 [3]. Bowen summarized the state of the discussion of the Stephan curve in 2023 [33].

Frequency and duration of sugar intake influence acid production by bacteria; therefore, a high sugar uptake frequency promotes prolonged acidic microenvironments. Furthermore, ecological shifts toward acidogenic and aciduric taxa increase the demineralization potential of a biofilm. It has also been demonstrated that a sugar-rich diet influences the oral microbiome and leads to an increased acid formation below a biofilm [34]. Finally, inter-individual variation in plaque ecology affects caries progression. This illustrates the complexity of caries and the challenges of simulating this process in-vitro. Even if cariogenic biofilms are replicated in a laboratory set-up, the results will vary between individuals [35]. The effect of biofilms (plaque) on the tooth surface is even more difficult to assess because this µm-thick layer of bacteria inside an extracellular matrix constitutes an efficient barrier for liquids and ions that separates saliva and enamel.

## 6. The Effect of Saliva

In general, tooth enamel is able to remineralize from saliva after acidic attack [8,28,36,37]. This is a continuous dynamic equilibrium between dissolving enamel and reforming enamel from saliva [38]. The balance between demineralization and remineralization is regulated by pH, ion concentrations (Ca^2+^, PO_4_^3−^, F^−^), and salivary flow. A disturbance of this equilibrium initiates a mineral loss. A lower salivary flow rate increases caries susceptibility due to decreased remineralization and pH-buffering. In particular, xerostomia (decreased saliva production) markedly elevates the caries risk by reducing neutralization, clearance, and remineralization. The presence of calcium ions and phosphate ions may slow down the dissolution of enamel, provided that the pH is not too low. Saliva is in continuous contact with the tooth surface (unlike acidic beverages), therefore its effect on teeth lasts much longer than that of food or beverages.

From a biological point of view, the composition of saliva is defined by pH, ion concentrations, bacterial load, and protein composition. This again leads to the conclusion that the situation in the mouth is very complex and highly variable between individuals. In a comprehensive literature review in 2024, Fu et al. pointed out that although there are synthetic formulations of saliva for in-vitro experiments, none of them include the entire variety of proteins that form the pellicle [39]. As the composition of saliva varies between individuals, the tendency for demineralization is also variable. Therefore, it is not possible to give a uniform critical pH value below which a demineralization will occur for an individual person.

**Figure 2:**
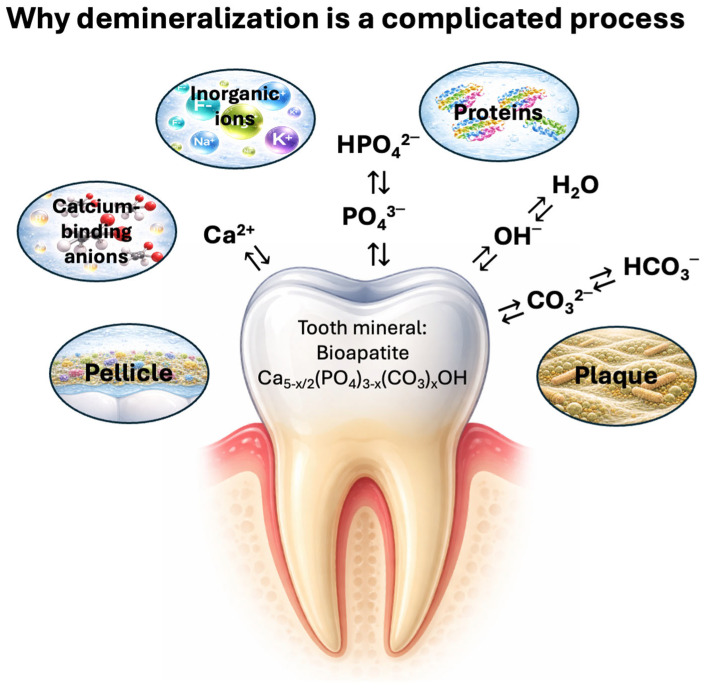
Factors that influence the demineralization of enamel.

Figure 2 gives a schematic view of the factors that come into play when the demineralization of enamel is considered.

## 7. In-Vitro Erosion Studies

It is not possible to study enamel demineralization on the nano-scale in-vivo. The underlying processes occur on a small length scale and the necessary analytical methods like electron microscopy, X-ray diffraction, nanoindentation, or elemental analysis are not applicable in the mouth. Therefore, many efforts have been devoted to investigating enamel demineralization in-vitro. In general, a demineralization occurs exclusively through acidic attack together with calcium-binding ligands like lactate, if present. This situation can be modelled in-vitro, usually by immersion of teeth into solutions with variable composition.

Erosion has been frequently replicated in-vitro, usually with cleaned teeth, i.e., without a pellicle. The applied pH is usually lower and the exposition time is usually longer than in the physiological situation in the mouth to achieve meaningful kinetic data within a suitable observation period. Of course, the acidic attack during drinking and eating is temporary and does not involve a continuous pH drop. This has been taken into account by dynamic pH cycling experiments that sometimes also replicate the remineralization from saliva between the acidic attacks. In general, erosion is easier to simulate than caries because no bacteria are involved, but the effect of proteins is usually neglected.

In-vitro experiments are usually performed with extracted human teeth, like third molars, or with bovine teeth. The latter are more easily accessible. As teeth have a curved surface that is sometimes impractical for experiments, they are often cut to form a planar surface that still exposes enamel only. There are some structural differences between human and bovine enamel, but in general they are considered sufficiently similar to use bovine teeth as models for human teeth [40]. However, Nancollas and Chen demonstrated differences in the dissolution rate between bovine and human enamel [19].

Von Fraunhofer et al. have studied the demineralization of human teeth during exposition to different commercial acidic beverages. The mass loss was measured gravimetrically during immersion for up to 336 h (14 d). The fact that it was 15 wt% for the most aggressive soft drink and in the other cases between 4 and 8 wt% indicates that erosion is still slow, as the exposition time during daily drinking is much shorter than 336 consecutive hours [2]. Inchingolo et al. published a systematic review in 2023 on the effect of carbonated soft drinks on enamel. Besides the well-known principles of erosion, they identified a limited salivary flow as a risk factor for erosion and also showed that the presence of calcium and phosphate can reduce erosion and enamel softening. However, a high concentration of some calcium salts can change the taste of the beverages, reducing their acceptance among consumers. Interestingly, sports participation was identified as a risk factor for erosive tooth wear due to a higher consumption of erosive drinks [41], alongside a vegetarian diet where no reason for the correlation was given [42].

Shellis et al. studied the demineralization of enamel by citric acid in the presence of calcium or phosphate ions. They found that calcium prevented demineralization if the pH was 3.25 or 4 but not at a pH of 2.5. In contrast, phosphate had no protecting effect in the pH range of 2.5 to 4 [43]. Chemically, it is reasonable to ascribe this observation to the fact that Ca^2+^ is present at any pH, whereas PO_4_^3−^ is mostly protonated at this low pH so that it scarcely contributes to the ion product, i.e., the supersaturation with respect to calcium phosphate.

Sintered hydroxyapatite with about 20% porosity was found to dissolve significantly slower at low pH than enamel, an observation that was ascribed to the presence of carbonate in enamel [44]. However, Chen and Nancollas have shown that the presence of carbonate does not affect the solubility of enamel at a pH of 4.5 [19].

## 8. The Kinetics of Enamel Dissolution by In-Vitro Studies

It is important to study the time-dependent demineralization of enamel to understand the underlying processes. This requires quantitative time-resolved methods. Major approaches are the measurement of released calcium and/or phosphate, the measurement of the pH, and the measurement of the consumed acid in the demineralization solution. These approaches give the integral extent of demineralization but no local information. Microanalytical methods (e.g., spectrophotometry, pH, calcium-ion selective electrodes) on a scale down to several nanoliters have been introduced by Vogel et al. to probe the local conditions during acidic attack on teeth [45].

It is usually assumed that enamel begins to dissolve below a pH of roughly 5.5 [33]. The dissolution accelerates with decreasing pH due to the increased proton concentration. The composition of the immersion medium changes during enamel dissolution. This affects the demineralization kinetics as the concentration of calcium ions and phosphate ions in the solution increases. Unless buffered, the pH will also increase. In principle, a saturated solution is obtained when the ion product reaches the solubility product. In a closed system such as the mouth, the dissolution rate of enamel will be fastest at the beginning until it stops near saturation. This makes kinetic studies difficult because the rate of dissolution is time-dependent. Patel and Brown showed already in 1975 that the solubility of enamel is variable and also depends on the proportion that has been dissolved. This underscores the complex nature of enamel, which is not a pure and well-defined inorganic salt [17].

Kinetic data on dissolution are convoluted by diffusion processes. Patel et al. have shown how this can be overcome by rotating the exposed enamel surface inside the surrounding solution [18], adapting the concept of a rotating disc electrode from electrochemistry [46,47]. They found that the kind of acid affected the dissolution rate after comparing acetic acid, benzoic acid, and salicylic acid at pH 4.5. They concluded that the formation of calcium complexes and the pK_a_ of the weak acid play important roles, also affecting the pH buffering and the binding of released calcium cations [18].

## 9. Constant Composition Methods and pH Cycling Approaches

The problem of non-stationary dissolution conditions can be overcome by using large volumes for immersion which may, however, reduce the sensitivity when it comes to measuring calcium concentrations. A better alternative is the application of constant-composition techniques where pH and ion concentrations are kept constant by titrators. Chen and Nancollas have introduced the constant-composition method into demineralization studies of enamel in 1986 [19].

In an experiment at constant pH (titration to pH-stat; citric acid at a pH between 2.45 and 3.9), Shellis et al. showed that dentin dissolves much faster than enamel, which in turn dissolved faster than compressed synthetic hydroxyapatite [48]. This illustrates the different solubilities of microcrystalline hydroxyapatite vs. microcrystalline carbonated apatite (enamel) and nanocrystalline carbonated apatite (dentin).

Wang et al. studied the dissolution of enamel at constant-composition at pH 4.5 and constant undersaturation. They found considerable differences between primary (deciduous) enamel and permanent enamel. Primary enamel dissolved about six times faster, which was ascribed to the different structure leading to different degrees of erosion on the nanoscale [49]. This was developed into a model of enamel dissolution, also supported by in-situ atomic force microscopy. This model was based on the assumption that nanoscale crystallites that remain after dissolution of the original crystals possess a higher resistance towards dissolution than larger crystals [50]. This was ascribed to the fact that small crystals are smaller than “critical etch pits”, which makes them metastable in terms of dissolution [51].

Extending the concept of constant-composition techniques, ten Cate and Dujisters introduced the pH cycling model in 1982. This permitted the study of the alternating processes of demineralization and remineralization in-vitro [52], as well as correlations with in-vivo results [53]. Buzalaf et al. published a review in 2010 on pH cycling models to investigate the effect of fluoride dentrifices [54].

Fu et al. published a comprehensive literature survey in 2024 in which they reviewed the in-vitro models in cariology mineralization research, covering the period from 2019 to 2023 (195 articles). Interestingly, there is a wide range of demineralization solutions that are applied to simulate erosion, ranging from pH < 1 to pH = 5.0. These induce more or less rapid demineralization with observation times between 15 s and 10 weeks, illustrating the conflict between a rapid and strong effect at low pH and a slower but more natural erosion at high pH. pH cycling experiments are frequently used to simulate demineralization and remineralization for up to three months [39].

## 10. Enamel Dissolution Studies: A Historical Perspective

Robinson et al. comprehensively reviewed current knowledge on enamel caries in 2000 [20]. They discussed the different stages of enamel dissolution by caries, and also the underlying chemical processes. At this time, most of the accumulated knowledge was based on physicochemical considerations, e.g., the diffusion of ions, the presence of more or less soluble calcium phosphate phases, and dissolution and reprecipitation equilibria. This was based on extensive in-vitro experiments that were carried out since the 1960s in order to describe and understand the underlying processes.

The derivation of fundamental kinetic equations on a physico-chemical basis started in the 1960s and peaked in the 1990s [20,44,55,56,57,58,59,60,61,62,63,64,65,66,67,68,69,70,71]. It is notable that in these in-vitro experiments very thorough dissolution experiments were carried out and kinetically interpreted but the influence of the organic material (e.g., pellicle, proteins in saliva) was almost always neglected. Thus, it is unlikely that the obtained kinetic data can be generalized to the actual situation in the mouth. Nevertheless, these experiments and computations gave important insight into the basic processes that occur during demineralization, which lays the basis for understanding this process under more complex conditions.

It is important to emphasize that many of these classical in-vitro experiments were conducted under controlled conditions, sometimes in constant-composition experiments [72]. In contrast, many experiments that were carried out later were conducted under non-stationary conditions where the resulting data are much more difficult to interpret. This shift in focus is due to the general realization that demineralization is too complex to be fully described by chemical equilibria and rate constants, given the presence of biomolecules. It also reflects the introduction of the increasingly sophisticated experimental methods that are discussed in the next section.

## 11. In-Vitro Erosion Studies with Spatial Resolution

A local view on the tooth during demineralization is required to get better insight into the underlying processes. Structure-sensitive methods to probe the tooth itself involve monitoring the tooth surface in-situ during demineralization, e.g., by atomic force microscopy (AFM) or ex-situ analysis, e.g., by electron microscopy, and monitoring the mechanical properties of enamel during and after demineralization. We can also distinguish between a two-dimensional analysis during demineralization, which gives a lateral view, and a three-dimensional analysis that gives a comprehensive image of at least a part of the volume of the whole tooth. X-ray microradiography is basically a one-dimensional method that gives information perpendicular to the tooth surface [55].

Besnard et al. published a very comprehensive review on the application of synchrotron radiation to study the structure and function of human enamel. They demonstrate how such high-end techniques were used to understand structure, demineralization, and remineralization on various length scales. Thematically, the techniques can be grouped into spectroscopy, diffraction, and tomography/imaging. The interplay between the enamel structure and the demineralization process can be studied. Cooperations between dentists and physicists can give remarkably deep insight, although the number of samples that can be analyzed at a synchrotron during beamtimes is limited and teeth are usually cleaned before the investigation to remove the biological material [9]. Fu et al. [73] and Chamard et al. [8] have summarized the currently available methods to study caries-induced de- and remineralization in two comprehensive reviews where the underlying processes and the nature of enamel are also described.

A powerful in-situ technique is AFM, which basically follows the surface roughness by scanning the surface with a very fine tip. It can also be used for indentation, giving mechanical properties of a sample surface. Barbour et al. studied the hardness of human enamel by nanoindentation during acidic attack at pH values between 2.3 and 6.3, using citric acid as the demineralizing agent for 120 s. They found an almost linear decrease in enamel hardness from 6.3 down to 3.1. Below that pH, the loss of hardness remained constant. The elastic modulus followed a similar trend. This indicates that the tooth structure is rapidly affected, e.g., during the consumption of acidic beverages, induced both by a low pH and the presence of the complexing citric acid [74]. Barbour et al. also showed that the presence of calcium ions during the acidic attack prevented the softening of enamel to some extent [75].

Voegel and Frank showed by transmission electron microscopy that enamel crystals dissolve during caries attack along the crystallographic *c*-axis (which is the long axis of the enamel crystal rods), leaving behind a partially hollow rod [76]. Salvati et al. used kinetic and structural finite element modelling to simulate the demineralization of enamel at the individual rod level. This was supplemented by high-resolution synchrotron microcomputer tomography (µCT) with a voxel size of 325 nm and focused ion beam-electron microscopy (FIB-SEM-STEM). A major observation was that the demineralization occurred first in the interrod regions [77]. Sui et al. performed an in-situ study at the synchrotron that combined small-angle X-ray scattering (SAXS) and wide-angle X-ray scattering (WAXS) during demineralization of a human tooth at pH 2.2 with lactic acid for up to 42 h. They could show how demineralization proceeds by dissolution of the enamel rods into the enamel structure [78].

Harper et al. studied the demineralization of enamel at pH 2.2 with lactic acid and performed µCT and light microscopy. They found that demineralization proceeds along the Hunter Schreger Bands (HSBs), which are superstructures inside enamel where enamel prisms cross [79].

Besnard et al. analyzed the structure of enamel via a combination of electron microscopy (FIB-SEM) and scanning X-ray diffraction at a resolution of about 500 nm. They showed a change in texture, i.e., of the orientation of the hydroxyapatite nanorods by X-ray diffraction after acidic etching, and different etching patterns of the hydroxyapatite rods [80].

Tsai et al. showed how enamel demineralization can be detected in-situ during attack by 37% phosphoric acid by optical coherence tomography (OCT) with a spatial resolution of about 2 µm. This is a non-destructive method that is based on the detection of back-scattered light, which provides information up to about 1 mm deep into the enamel. It was demonstrated that the internal structure of enamel was eroded within a few seconds after acid application [81]. Terahertz-based scanning near-field optical microscopy (s-SNOM) can give nondestructive insight into tooth enamel, also after demineralization, with a lateral resolution of about 100 nm and a penetration depth of several micrometers [82].

Baumann et al. investigated the effect of the incorporated proteins inside enamel that remained after eruption, i.e., the structural proteins that remained after biomineralization. Enamel was deproteinated with hydrazine and then subjected to acidic erosion at pH 3.6. It was found that deproteinated enamel became softer and dissolved faster than untreated enamel. Although, the effects were statistically significant, they were not very strong, showing that the incorporated proteins give only low protection against acidic attack [83].

Recently, He et al. have shown how a machine learning system was successfully trained to recognize early-stage caries based on intra-oral photographs. However, a considerable amount of training data will be required before this method can be applied more broadly [84].

## 12. In-Vitro Demineralization Studies in the Presence of Proteins or Bacteria

Demineralization experiments that are carried out under more biological conditions are rare. The oral milieu has been simulated in-vitro by installations denoted as “Artificial Mouth”. Basically, these are more or less sophisticated bioreactors where bacteria are cultivated on teeth for days and weeks under a continuous supply of nutrients. Tang et al. reviewed the historical development that started in the late 19th century [85]. Today, the very first installations would not be even be called “instruments”, being just simple set-ups to assess tooth dissolution. The first continuous-flow system, in which the anti-caries agents from toothpastes were also studied [86], was presented by Pigman et al. in 1952 [87].

Improvements in sterility, nutrient control, microbial habitats, and artificial saliva compositions led to functional bioreactors at the end of the 20th century. Exterkate et al. introduced the *Amsterdam Active Attachment biofilm model* (AAA) in 2010, where biofilms were cultivated in-vitro first in cell culture dishes [88] and later on enamel discs [89]. In a recent study, Amaechi et al. demonstrated how the natural caries process can be simulated in an artificial mouth with *S. mutans*, which formed white spot lesions on human teeth within a few days [90]. A complimentary technique are in-situ studies where teeth or other samples are introduced into the mouth of humans to assess their change in contact with saliva and the oral microbiome [91]. These form a bridge between in-vitro and in-vivo experiments, although inter-individual variations require large sample numbers [92]. Note that the term “in-situ” in dentistry must not to be confused with the term “in-situ” in chemistry and physics, where it denotes the measurement of properties during a dynamic process.

## 13. Caries and White Spot Lesions

A white spot lesion is a subsurface porosity beneath an intact surface layer and an early indicator of demineralization by caries [93]. White spot lesions are easily recognized by dentists due to their optical appearance, due to a change in optical refraction. White spot lesions are formed below cariogenic biofilms and precede caries cavitation. They frequently accompany fixed orthodontic treatments, e.g., brackets that are glued to the tooth surface. Prada et al. reviewed the literature in 2024 with respect to their treatment by resin infiltration. They found that there is a high prevalence of white spot lesions during and after orthodontic treatment (more than 50% and up to 97%), although the incidence is already high before an orthodontic treatment (15% to 40%). As major cause, the difficulty of removing the plaque around and below the orthodontic appliance was identified [94].

From the viewpoint of chemistry, the occurrence of white spot lesions is a peculiar fact. In general, one would expect the acid-induced dissolution of calcium phosphate to start at the surface, forming a hole like an open quarry. Obviously, there is a mechanism that protects the outer layer of enamel from initial dissolution. This could be due to gradients in structure and/or composition from the tooth surface towards its interior. White spot lesions have therefore extensively been investigated by in-vitro experiments where human or bovine teeth were exposed to acids in the presence and in the absence of biomolecules.

The exact protection mechanism during the formation of white spot lesions is unknown, but it is likely related to the attached proteins, which buffer the acidic attack and protect the structure of the calcium phosphate. The thickness of the solid cover layer of a white spot lesion has been measured at about 70 µm [59], protecting a cavity of about 100–200 µm in depth and a lateral size of several mm^2^. As a protecting agent in in-vitro studies, hydroxyethyl cellulose (HEC) [57] and disodium methanehydroxydiphosphonate (MHDP) [59] have been proposed as models of the pellicle as early as 1966 and 1979, respectively.

Anderson and Elliot introduced real-time microradiography in 1992 to investigate the dissolution of enamel during acid attack (time resolution several hours, spatial resolution about 10 µm). This method is based on X-ray absorption by the highly mineralized enamel [55]. It has been demonstrated how white spot lesions develop and form a hollow layer below an intact enamel surface. Anderson and Elliot concluded that this process is complex and cannot be assigned to one single cause. They also showed that such a subsurface demineralization can also occur in other systems like Ca(OH)_2_ and Mg(OH)_2_ at the correct conditions, i.e., it is not unique to enamel [55].

Gao and Anderson investigated the subsurface demineralization by microradiography under constant-composition conditions, keeping the ion concentrations and the pH constant. They were also able to replicate the subsurface demineralization of white spot lesions with acetic acid at pH 4 and found that the presence of gelatin led to some degree of remineralization. They derived extensive kinetic data and concluded that the dissolution process of enamel and not the transport of ions is rate-limiting [65,95].

Gray provided a kinetic equation to describe the dissolution rate of enamel based on in-vitro studies, and argued that the diffusion of undissociated acid through enamel is responsible for the formation of white spot lesions [57]. This was confirmed by Featherstone et al., who in 1979 proposed a stable surface layer on top of a white spot lesion that acts as diffusive barrier, protected by the pellicle [59].

Margolis et al. studied demineralization by lactic acid, acetic acid, and propionic acid at a pH of 4.3 to 6.0 by microradiography, derived equations for the demineralization kinetics, and also found subsurface lesions. Notably, demineralization was faster with lactic acid than with acetic acid and propionic acid, pointing to a complexation of calcium that enhanced dissolution [96]. Anderson and Elliot compared the rate of mineral loss in enamel by microradiography perpendicular and parallel to the tooth surface. Interestingly, the rate perpendicular to the surface was about 14% higher than that parallel to the surface, and faster toward the interior of the tooth. This suggests that the anisotropy of enamel makes it more resistant against acidic attack from the surface than from within, e.g., from a caries lesion [56]. Amaechi discussed the idea of treating caries via remineralization in 2017, and recommended that any remineralization treatment should be accompanied by general behavioral modification in oral health by the patients [97].

The coupled diffusion of acid into the enamel and dissolution products out of the lesion has been suggested to explain the formation of subsurface lesions. In a comprehensive study at variable electrolyte concentration at pH 4.6, Anderson et al. showed that this diffusion process cannot be solely responsible for the formation of such white spot lesions [44]. However, from a chemical viewpoint, the postulation of H_3_PO_4_ and Ca(OH)_2_ as neutral components that diffuse as one species is puzzling and, given the dissociation of these compounds in water, not correct [44].

Robinson et al. proposed that the formation of a poorly soluble fluoride-rich layer is responsible for the remaining enamel layer of a white spot lesion. They also argued that the presence of magnesium- and carbonate-rich phases leads to faster dissolution. A number of solid phases were proposed; however, these were never identified. The effect of the (bio)molecules in saliva, pellicle, and plaque was only briefly considered, although it was conceded that they probably play an important role. Finally, Robinson et al. proposed that the treatment of caries lesions with a high local dose of fluoride could lead to their repair [20].

Robinson et al. also proposed that the pellicle protein layer can act as semipermeable membrane that permits the influx of acid but constrains the efflux of ions created by the dissolution of enamel [20]. Thus, these ions meet a diffusion barrier below the pellicle, where they reprecipitate to form a solid cover of the underlying cavity. This also follows Robinson et al.’s observation that the cover layer does not consist of the original enamel but of reprecipitated mineral and undergoes a continuous restructuring of dissolution and reprecipitation [20]. Flemming et al. have also argued that the formation of white spot lesions is the results of the pellicle that prevents mineral dissolution and therefore preserves a thin layer above the lesion cavity [25].

Al-Obaidi et al. studied human enamel subsurface lesions that were created by pH cycling between pH = 4.3 (demineralization solution with acetic acid) and pH = 7 (remineralization solution with calcium and phosphate) for 24 h in each cycle without any organic/biological material. For comparison, they studied natural teeth that had developed white spot lesions. Confocal Raman microscopy based on the absorption band of phosphate showed an intact surface layer with a thickness of several micrometers. The thickness of the lesion, i.e., the hollow space below the surface layer, was about 10–20 µm for artificially created lesions and 30–40 µm for natural lesions [98].

In 2012, Ilie et al. presented a very comprehensive numerical model that simulates enamel demineralization in the presence of plaque (representing four bacterial species), taking into account the diffusion of ions, acid-base equilibria, sugar consumption and microbial conversion by bacteria, and the demineralization of enamel. They could demonstrate how a 2 min pulse of glucose in saliva, equivalent to the consumption of food or beverage, leads to a pH drop within a few minutes, associated with a rapid drop in pH in the biofilm down to 4.5 to 5. Although glucose is consumed after about 20 min, it takes about 40 min for the pH in the biofilm to increase above 5.5 (critical pH for demineralization) and about 140 min to return to the initial value of 6.5. They also showed that the negative effect of acidification was larger if the plaque was thicker, highlighting the beneficial effect of dental hygiene. Furthermore, they demonstrated that one short intake of sugar is much less destructive than a continuous exposure (“slow social drinking”). In that case, the pH inside the biofilm remains continuously below the critical pH value because sugar is continuously delivered and the pH cannot rise above the critical value [99]. In 2014, this model was extended to include remineralization during pH cycling induced by up to 80 doses of glucose, followed by resting periods for remineralization from saliva. Although it is unlikely that remineralization can actually be numerically considered with a simple rate equation (nucleation and other calcium phosphate phases all play a role [28]), it was convincingly shown how a cyclic demineralization-remineralization process in the presence of plaque (only one bacterial species) can lead to white spot lesions. They concluded that a white spot lesion requires pH cycling and the presence of plaque, again with a thicker plaque inducing more white spot lesions. The presence of fluoride at the surface of enamel also contributed to the formation of white spot lesions, supposedly due to the lower solubility of fluoroapatite. However, as the white spot lesions were also found in the simulations without taking fluoride into account, the effect of fluoride appears to be supporting but not the only case [100]. These two landmark publications explained many processes that were previously accessible only through experimental studies. It is remarkable that 34 citations for ref. [99] and 12 citations for ref. [100] indicate that they have not (yet?) received the attention that they deserve.

To conclude this section, extensive research over the past sixty years has advanced our understanding of white spot lesions, particularly highlighting the role of weak organic acids (such as lactic and acetic acid) from cariogenic biofilms in subsurface enamel dissolution. These acids can diffuse through the enamel in an undissociated form and release protons beneath the surface, leading to demineralization. The organic pellicle plays a protective role, allowing demineralization to occur beneath an intact outer enamel layer, resulting in white spot lesions. Despite its importance, the pellicle remains difficult to study due to its dynamic nature and constant interaction with saliva, making processes like ion and protein diffusion into lesions hard to measure precisely. The simulations by Ilie et al. demonstrated that the interplay between plaque and enamel is responsible for the demineralization, and that pH cycling is necessary to form a protective cover on top of the lesion.

## 14. Bibliographic Study on Enamel Demineralization

Even after almost a century of research, enamel demineralization is still a widely investigated research topic. This is demonstrated here by a short bibliographic study that is not intended to be comprehensive but to illustrate the focus of research over the last 70 years. Table 1 gives the major results. The general search term (“tooth” OR “teeth” OR “dental”) to capture most publications dealing with dentistry shows the normal increase in publications over the years. The interest in demineralization increased in the 1980s but accounts for only 1.1% of all publications concerned with dentistry. The effects of saliva, pellicle, or plaque on demineralization comprise about one fifth of this subgroup, increasing in the 1990s. Within the subgroup of demineralization, 39 publications contain the keyword “synchrotron” and 84 publications contain the keyword “radiography”. Among all dentistry-related references, erosion accounts for about 1% whereas caries is much more prominent with 10.5%. Research on white spot lesions became more prominent in the 1980s, whereas the structure of human enamel has been explored since the 1970s.

These data reflect the strong interest in dentistry in general and in caries in particular. Demineralization was recognized as an increasingly important topic in the 1980s but appears to remain underrepresented. Notably, the search term (“apatite” AND “solubility product”) gave 221 hits with almost constant yearly numbers from 1986, underscoring that this topic is still a matter of discussion and research.

## 15. The Role of Caries-Preventing Agents

Preventing or even reversing enamel demineralization is probably the most important challenge in dentistry. We will briefly discuss some current concepts that are based on chemical agents. For a more comprehensive discussion on remineralization, the reader is referred to an earlier review article [28].

The protecting effect of fluoride against caries has been well established in dentistry for decades [101]. Consequently, the role of fluoride during demineralization has been studied and discussed extensively (see, e.g., refs. [102,103]). It has frequently been claimed that the outer layer of a tooth contains enough fluoride to slow down the dissolution under acidic attack. This is based on the assumption that the incorporation of fluoride into hydroxyapatite leads to the formation of fluoroapatite with a lower solubility. Another frequent claim is the formation of a protective insoluble layer of calcium fluoride, i.e., fluorite, CaF_2_, that acts as acid-resistant coating on the tooth surface [104].

Unfortunately, we cannot follow the argument of a protecting fluoride-rich layer, in line with objections that were raised already decades ago, presented, e.g., by Fejerskov in 2004 [105] and summarized as part of a personal review by ten Cate in 2015 [106]. The concentration of fluoride in and on tooth enamel is always too low to prevent dissolution under acidic attack. This was demonstrated convincingly as early as 1988 by Ogaard et al., who showed that shark teeth, whose enamel(oid) consists of stoichiometric fluoroapatite [107], dissolve almost as fast as human teeth consisting of hydroxyapatite under acid attack [108].

Furthermore, a solid layer of fluoroapatite or of calcium fluoride was never detected, nor is it likely to be thick enough to act as a passivating layer that prevents acidic corrosion [109,110,111,112]. Such a layer has been claimed but never proven by X-ray diffraction, which would be the only suitable method of detection. The solubilities of fluoroapatite and CaF_2_ are not much different from hydroxyapatite. Thus, even a thick layer of these compounds would not increase the stability against acids.

Consequently, there is no easy way to explain via chemical solubility why fluoride protects enamel against acidic demineralization. It is likely that its protective effect is related to a faster remineralization of teeth from saliva. This was pointed out by Larsen, who proposed that fluoride in solution (i.e., in saliva) is more important against enamel dissolution than fluoride incorporated into enamel [103]. ten Cate concluded that fluoride is not active by protecting against demineralization but by enhancing remineralization, e.g., by promoting nucleation [106]. Buzalaf et al. published a review in 2010 on pH cycling models to investigate the effect of fluoride-containing toothpastes on erosion, underlining these assumptions [54]. A discussion on the effect of fluoride on caries prevention can be found in an earlier review [111].

The dissolution of fluoridated apatite leads to the release of fluoride ions, F^−^. Fluoride ions may be protonated to give the toxic compound hydrogen fluoride (or hydrofluoric acid), but as the pK_a_ value of HF is 3.17, it will appear only in significant amounts below a pH of about 3.5, which is unlikely in the mouth [113]. In any case, most fluoride in the mouth will come from toothpaste and not from demineralized enamel, given the low amount incorporated into enamel.

Calcium phosphate particles, which are used in some toothpastes, may enter cavities in enamel [114,115]. Hydroxyapatite particles have been shown to interact with the enamel surface and to fill superficial defects [116]. They undergo the same dissolution process as tooth mineral, but their presence on the enamel surface can increase the local concentrations of calcium and phosphate and thereby constrain the extent of dental erosion [117,118] and caries [116]. In toothpastes, they can help to remineralize and to restore enamel after attack by acids. Owing to its high specific surface area, particulate hydroxyapatite can dissolve faster than enamel mineral, thereby shifting the overall equilibrium between demineralization and remineralization towards remineralization [119].

To mention a more exotic but nevertheless interesting approach to preventive dentistry from biology, the application of phages, i.e., viruses that attack bacteria, in the mouth is an interesting idea that is in line with phage therapy to combat bacterial infections and biofilms [120,121,122]. As the oral microbiome is very complex and also houses a community of phages and bacteria [123], this possibility has not yet been explored, also because of the high specificity of phages for individual bacterial strains. In the future, the idea of combatting bacteria with phages may be an interesting option.

## 16. Conclusions

**Figure 3:**
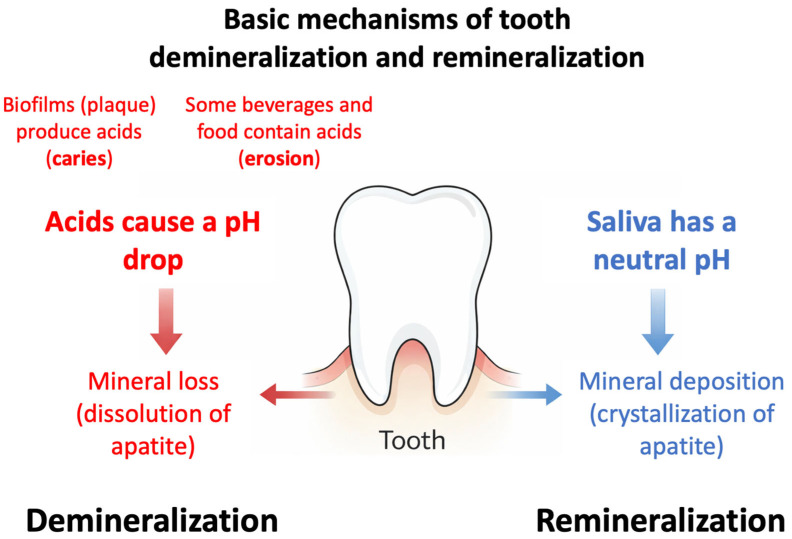
Fundamental processes that govern the equilibrium enamel demineralization and remineralization.

Demineralization can be caused by bacteria in biofilms (plaque) that cause caries or by acid-containing food and beverages that cause erosive tooth wear. The origin of the acid plays a minor role; the drop in pH is decisive. Remineralization from saliva can restore the lost mineral in many cases. Figure 3 summarizes all these processes.

In healthy individuals, demineralization and remineralization are generally in dynamic equilibrium, maintaining the integrity of enamel. Nevertheless, the underlying mechanisms are still not fully understood, despite decades of experimental and theoretical efforts. Both processes are in principle governed by the solubility product of hydroxyapatite. When the ion product of calcium and phosphate in saliva exceeds the solubility product, the solution is supersaturated and precipitation of calcium phosphate mineral is thermodynamically favored, whereas undersaturation leads to dissolution of enamel. However, in the oral cavity, these processes are far more complex than is suggested by this simple thermodynamic description. Saliva contains many components, including inorganic ions, organic complexing agents, and biological macromolecules like proteins, which all influence the demineralization.

Therefore, enamel is never exposed to a simple aqueous solution of inorganic ions. Seconds after cleaning, the tooth surface is covered by a protein pellicle, which is subsequently colonized by microorganisms that form a bacterial biofilm within hours. Consequently, the enamel surface is constantly immersed in a dynamic biological environment consisting of saliva, biomolecules, and microbial communities, which strongly modulate the local chemical conditions that govern demineralization.

Demineralization is therefore a multifactorial but partially reversible process that depends on acid-base equilibria in saliva and biofilms, diffusion processes within the enamel, and the interaction of mineral phases with biomolecules and microorganisms. A major challenge in studying these processes is that they occur on micro- and nanometer length scales under highly complex and dynamic biological conditions that cannot be probed directly in-vivo and are difficult to reproduce in-vitro. The necessity (or desire) to induce the process within a reasonable time span for an experimental study often leads to experiments at unrealistically low pH. Furthermore, it is very challenging to replicate the biological environment in the oral cavity during in-vitro experiments. As a result, a comprehensive mechanistic understanding of enamel demineralization and remineralization is still needed, although simulations have shed some light onto the underlying mechanisms.

Future in-vitro research should involve demineralization in the presence of proteins and bacteria under controlled conditions, i.e., at least with controlled pH, during practically relevant times (hours to days), and ideally with pH cycling. An extension of modelling approaches to more complex systems and on smaller length scales, including proteins besides organic and inorganic compounds and bacteria in plaque, will also give more insight into processes that cannot be directly monitored. The individual variability of the oral microflora makes general conclusions complicated, but can probably be better addressed in the light of ongoing proteomics research and its correlation with oral health. We must acknowledge, however, that to comprehensively understand the process of demineralisation, there is still a long way to go. First, this is due to the many factors of interest that are not even known in many cases, like the whole range of ion–protein–enamel interactions. Second, the lack of analytical methods that can be applied in-situ in the oral cavity to elucidate processes that occur on the nanoscale (like electron microscopy) limits our ability to understand the processes that occur on the enamel–saliva interface.

## Figures and Tables

**Table 1:** Bibliographic study in the Web of Science as of 23 March 2026, searching within all entries from 1946 to 2025 the field “topic = TO”. No wildcards were used. The results were clustered over five-year periods.

Year Range	“Tooth” OR “Teeth” OR “Dental”						“White Spot Lesions”	“Human” AND “Enamel” AND “Structure”
	All	AND “Demineralization”			AND “Erosion”	AND “Caries”		
		All	AND “Saliva”	AND (“Pellicle” OR “Plaque”)				
2016–2025	199,782	2413	441	387	2135	22,673	1488	776
2006–2015	99,131	1296	216	257	1283	9991	490	542
1996–2005	54,603	599	100	153	646	5168	219	254
1986–1995	28,774	279	34	88	163	2588	112	105
1976–1985	19,307	93	6	11	56	1673	63	82
1966–1975	9113	13	0	0	19	720	10	24
1956–1965	3506	8	1	0	10	467	10	8
1946–1955	3013	7	0	0	16	607	6	8
Total	417,229	4708	798	896	4328	43,887	2398	1799
	100%	1.1%			1.0%	10.5%		
		100%	17%	19%				

## Data Availability

No new data were created or analyzed in this study. Data sharing is not applicable to this article.
